# Therapeutic potential of hair follicle-derived stem cell intranasal transplantation in a rat model of ischemic stroke

**DOI:** 10.1186/s12868-022-00732-w

**Published:** 2022-07-25

**Authors:** Seyedeh Maryam Mousavi, Bijan Akbarpour, Saeideh Karimi-Haghighi, Sareh Pandamooz, Ivaldo Jesus Almeida Belém-Filho, Marianella Masís-Calvo, Haniye Salimi, Ramin Lashanizadegan, Alireza Pouramini, Maryam Owjfard, Etrat Hooshmandi, Mahnaz Bayat, Seyedeh Shaghayegh Zafarmand, Mehdi Dianatpour, Mohammad Saied Salehi, Afshin Borhani-Haghighi

**Affiliations:** 1 Department of Basic Sciences, Faculty of Veterinary Medicine, Kazerun Branch, Islamic Azad University, Kazerun, Iran; 2 Clinical Neurology Research Center, Shiraz University of Medical Sciences, Shiraz, Iran; 3 Stem Cells Technology Research Center, Shiraz University of Medical Sciences, Shiraz, Iran; 4 Department of Pharmacology, School of Medicine of Ribeirão Preto, University of São Paulo, Ribeirão Preto, São Paulo, Brazil; 5 School of Biology, University of Costa Rica, San Jose, Costa Rica; 6 Transplant Research Center, Shiraz University of Medical Sciences, Shiraz, Iran

**Keywords:** Cerebral ischemia, Cell therapy, Epidermal neural crest stem cell, EPI-NCSCs, Nasal route

## Abstract

**Background:**

Stem cell-based therapy has received considerable attention as a potential candidate in the treatment of ischemic stroke; however, employing an appropriate type of stem cells and an effective delivery route are still challenging. In the present study, we investigated the therapeutic effect of safe, noninvasive, and brain-targeted intranasal administration of hair follicle-derived stem cells (HFSCs) in a rat model of ischemic stroke.

**Methods:**

Stem cells were obtained from the adult rat hair follicles. In experiment 1, stroke was induced by 30 min middle cerebral artery occlusion (MCAO) and stem cells were intranasally transplanted immediately after ischemia. In experiment 2, stroke was induced by 120 min MCAO and stem cells were administered 24 h after cerebral ischemia. In all experimental groups, neurological performance, short-term spatial working memory and infarct volume were assessed. Moreover, relative expression of major trophic factors in the striatum and cortex was evaluated by the quantitative PCR technique. The end point of experiment 1 was day 3 and the end point of experiment 2 was day 15.

**Results:**

In both experiments, intranasal administration of HFSCs improved functional performance and decreased infarct volume compared to the MCAO rats. Furthermore, NeuN and VEGF expression were higher in the transplanted group and stem cell therapy partially prevented BDNF and neurotrophin-3 over-expression induced by cerebral ischemia.

**Conclusions:**

These findings highlight the curative potential of HFSCs following intranasal transplantation in a rat model of ischemic stroke.

## Introduction

Stroke, a cerebrovascular disorder, is considered one of the main causes for mortality and disability all around the world [1]. Although current managements, such as mechanical thrombectomy and administration of tissue plasminogen activator have transfigured the ischemic stroke treatment, these approaches have been limited due to several disadvantages, such as treatment failure, risk of hemorrhage, and narrow time window [2]; therefore, finding new alterations is prime importance.

Stem cell-based therapy, represents a promising approach for the treatment of ischemic stroke owing to angiogenesis and neurogenesis induction potential as well as neuroprotective and immunomodulatory properties [3]. Up until now different cell types such as embryonic stem cells, neural stem cells, mesenchymal stem cells, induced pluripotent stem cells, along with vascular and endothelial progenitor cells have been employed to treat animal models of stroke [4]. Although the beneficial potential of these cells has been demonstrated in preclinical investigations of cerebral ischemia, their applications involves several challenges and limitations, such as raising ethical concerns, possible tumorigenicity, requiring genetic manipulation, abundancy and accessibility [5]. Hence, using an easily accessible cell type ontologically related to the nervous system, with the potential to differentiate into neuronal/glial cells without raising ethical concern is more desirable.

Hair follicle-derived stem cells (HFSCs), also known as epidermal neural crest stem cells are reside of the embryonic neural crest, located in the bulge area of adult hair follicles [6]. These types of stem cells possess several benefits such as accessibility, abundancy, and high plasticity. They can be easily harvested from the hairy skin with a minimal invasive procedure [6]. Also, they have a potential to differentiate into a various cells, such as glial cells [7], neurons [8], as well as osteocytes and melanocytes [9]. Isolation of hair follicles from the skin does not raise ethical concerns and by autologous transplantation, immunological graft rejection would be avoided. In addition, up to now, no immunological rejection are reported following allograft or xenograft transplantation of HFSCs in several nervous system conditions such as Alzheimer’s disease [10], vascular dementia [11], peripheral nerve injury [12], also animals [13] and organotypic [14, 15] models of spinal cord injury. Moreover, HFSCs express a wide range of extracellular proteases, angiogenic factors, and growth factors that eventually may lead to neuroprotection and neo-vascularization [16]. Accordingly, HFSCs are considered as an attractive cell type that can be used in regeneration medicine.

Besides the stem cell type, the route of administration is also considered as one of the most fundamental prospects of cell therapy. Intravenous, intra-arterial, and intracranial routes are widely used to deliver stem cells to the brain following cerebral ischemia [17]. Entrapment of cells in the peripheral organs like the kidney, spleen, lung, and liver before they reach the brain is a major limitation of intravenous delivery [18]. Although intra-arterial administration led to a greater diffusion and distribution of stem cells into the lesion site, a higher incidence of microembolization along with cerebral blood flow reduction are still challenging [19, 20]. Also, intracranial transplantation is a highly invasive procedure that may inflict damage to the adjacent brain areas [17].

Therefore, employing alternative transplantation routes that can effectively penetrate the brain with minimal invasiveness and systemic exposure is of paramount necessity. In 2009, it has reported that cell transplantation through the intranasal route can reach the brain [21] and subsequent investigations during the last decade revealed intranasal administration of stem cells can be a feasible strategy in the treatment of central nervous system diseases [22]. In this regard, the present study was designed to evaluate therapeutic effects of intranasally applied HFSCs, as a safe and brain-targeted approach in a rat model of ischemic stroke. In doing so, neurological performance, short-term spatial working memory and infarct volume were assessed up to 14 days after cell therapy. In addition, relative expression of major neurotrophic factors in the ipsilateral striatum and cortex were evaluated.

## Material and method

### Animals and ethic statement

In the present study, experimental procedures were implemented on 72 Sprague-Dawley male rats, weighing 230–270 g, housed in a pathogen-free environment and standard cages under controlled conditions with free access to standard food and water, and kept on wood shavings throughout the whole study. External factors were maintained stable within approved limits and included a 12 h light/dark cycle, 50–60% relative humidity, and temperature between 20–22 °C. This experiment was approved by the Animal Care Committee of Shiraz University of Medical Sciences (IR.SUMS.REC.1400.514), all the methods was in accordance with the declaration of Helsinki and the study is reported in accordance with ARRIVE guidelines.

### The isolation and culture of HFSCs

The HFSCs were isolated from the bulge of rat hair follicles using the method described previously [23, 24]. In brief, the hair bulges were mechanically dissected from the whiskers of 4 rats and explanted on the collagen-coated 4-well plates. The hair bulges were cultured in minimum essential medium alpha (ShellMax, # M4140) containing 10% fetal bovine serum (FBS, Bio Idea, # BI1201), 10% day-11 chick embryo extract, and 1% penicillin/streptomycin (ShellMax, # P3790) and incubated at 37 °C with 5% CO_2_. Seven days after migration, stem cells were passaged and HFSCs at passage 3 were used for transplantation.

### Verification of HFSCs

#### Immunostaining

Identity of migrated HFSCs was assessed by immunostaining against nestin as a neural crest stem cell marker and SOX10 as a neural crest cells marker [6] using mouse anti-nestin (1:50; Abcam, #ab6142) and rabbit anti-SOX10 (1:100; proteintech, 10422-1-AP) primary antibodies. Briefly, cells at passage 1 were seeded in a 4-well chambered cell culture slide and fixed with 4% paraformaldehyde. Following several washing steps, cells were blocked with 10% normal goat serum, 1% FBS and 0.1% Triton X-100 prepared in phosphate-buffered saline (PBS). Then, the primary antibodies were applied overnight at 4 °C. After washing cells and re-blocked with 3% bovine serum albumin for 10 min, cells were exposed to goat anti-mouse IgG AlexaFluor488 (1:1000, ThermoFisher, #A-11001) or goat anti-rabbit IgG AlexaFluor488 (1:1000, Abcam, #ab150085) secondary antibodies at room temperature for two hours. To counterstain the nuclei, the ProLong™ Glass Antifade Mountant with NucBlue™ Stain (Invitrogen, # P36985) was used to cover the chambers. Finally, immunofluorescent images were obtained using a Leica DM5000B epifluorescence microscope.

#### Immunophenotyping

To evaluate cell surface markers expressed by HFSCs and their purity, flow cytometry analysis was performed. In brief, HFSCs at passage 3 were blocked in PBS supplemented with 10% FBS for 20 min. Then, cells were incubated with CD34-PE, CD44-FITC, CD45-FITC and CD90-PerCP antibodies for 30 min at 4 °C. After washing steps, fluorescence was measured with a BD FACSCalibur.

##### Differentiation potential

The adipogenic and osteogenic differentiation potential of HFSCs were assessed after 21 days sub-cultured in specific differentiation culture mediums followed by Oil Red or Alizarin Red staining as described in detail before [25].

### Experimental design

#### Experiment 1

In experiment 1, rats were randomly assigned to 3 main groups (n = 10–12/group): (1) SHAM, experienced surgical procedures similar to other groups without middle cerebral artery occlusion (MCAO) and stem cell administration; (2) MCAO, underwent 30 min MCAO and received PBS intranasally immediately after recovery from the surgery; (3) rHFSC-IN, experienced 30 min MCAO and received HFSCs through the intranasal route immediately after recovery from the surgery. The end point of experiment 1 was day 3 after the surgery.

#### Experiment 2

In experiment 2, animals were randomly assigned to 3 main groups (n = 12/group): (1) SHAM, experienced surgical procedures similar to other groups without MCA occlusion and stem cell transplantation; (2) MCAO, underwent 120 min MCAO and received PBS intranasally one day after the surgery; (3) rHFSC-IN, experienced 120 min MCAO and received HFSCs through the intranasal route one day after the surgery. The end point of experiment 2 was day 15 after the surgery.

#### MCAO procedure

Experimental animals were subjected to transient MCAO as described before [26, 27]. Briefly, animals were anesthetized with subcutaneous injection of ketamine (100 mg/kg) and xylazine (5 mg/kg) mixture [28]. Following ligation of right common carotid artery and right external carotid artery, a silicone rubber-coated monofilament (#403556, Doccol Corporation) was entered into the right common carotid artery and gently advanced to occlude origin of MCA. Throughout the surgical procedure, Laser Doppler (ML191, AD Instrument, Australia) was used to monitor blood flow reduction. Besides, a heating pad and heating lamp were employed to maintain the rectal temperature at 37 °C. After 30 or 120 min of occlusion, the monofilament was carefully removed to enable reperfusion. Both MCA occlusion times have been chosen based on a comprehensive study conducted by Popp et al. who showed that 30 min MCAO resulted in mild stroke in rats; while 120 min MCAO led to moderate to severe stroke [29].

#### Transplantation approach

Immediately after recovery from the surgery (experiment 1) or 1 day after the surgery (experiment 2), each nostril was treated with 5 µl hyaluronidase (10 mg/ml, Sigma-Aldrich) prepared in PBS to increase the permeability of the nasal mucosa [30]. Thirty minutes later, 2 × 10^6^ HFSCs suspended in 100 µl PBS, were gradually administrated through the intranasal route in awake rats. MCAO rats just received hyaluronidase treatment.

#### Functional tests

In experiment 1, neurological deficits were assessed before the induction of ischemia (day 0), also 1 and 3 days post-surgery/cell therapy. In this regard, functional outcome was ranked according to the following scales, No = 0: lack of neurological impairment; Mild = 1: left forepaw disability; Moderate = 2: counterclockwise circling; Severe = 3: falling to the left; and unconsciousness = 4: lack of consciousness without spontaneous walking [27].

Our preliminary findings showed that the scoring method that has been used in the experiment 1, was not able to detect neurological deficits in the MCAO rats of experiment 2 after 15 days. Therefore, in the experiment 2, before the induction of ischemia (day 0), also one day after the surgery (prior to stem cell transplantation), neurological deficits were assessed by scoring method. Fourteen days after stem cell therapy, neurological performance was evaluated by cylinder rearing test as described in detail earlier [31]. Percentage of paw preference during full rearing was calculated as $\left[ {\left( {{\text{unimpaired right paw}}{-}{\text{impaired left paw}}} \right)/\left( {{\text{total forelimb usage}}} \right)} \right] \times 100$ [32].

#### Infarct volume measurement

At the end point of both experiments, rats were killed under deep carbon dioxide anesthesia, brains quickly removed and 2 mm coronal sections obtained by a brain matrix. The sections were stained with 0.5%, 2,3,5-triphenyltetrazolium chloride (TTC, Sigma) prepared in normal saline for 30 min at 37 °C. Due to the reduction of TTC to formazan by mitochondrial enzymes, viable brain regions were stained dark brick red; while necrotic and non-viable brain regions were unstained and remained white. Infarct volume was calculated using ImageJ software [24].

#### Body weight and survival rate

Body weight was measured before the surgery, one day after the surgery as well as at the end point of both experiments as a general marker for well-being. Moreover, mortality rate was calculated in all experimental groups.

#### Short-term memory evaluation

The Y-maze test was performed at the end point of both experiments to evaluate the short-term spatial working memory [33]. Total number of arm entries and sequence of entered arms were recorded for 8 min. Alternate arm return (AAR) and spontaneous alternation performance (SAP) were calculated by following formula:

$$\% {\text{AAR}} =\, \left[ {\left( {{\text{total number of alternate arm returns}}} \right)/\left( {{\text{total number of arm entries}} - 2} \right)} \right] \times 100.$$

$$\% {\text{SAP}} =\, \left[ {\left( {{\text{total number of spontaneous alternations}}} \right)/\left( {{\text{total number of arm entries}} - 2} \right)} \right] \times 100.$$

#### RNA isolation, cDNA synthesis, and target genes expression

In experiment 1, 3 days after surgery/stem cell therapy, 4–6 animals in each group were put to death under deep anesthesia. Brains were removed and two ipsilateral regions, striatum and cortex dissected and snap-frozen. Total RNA was extracted by YTzol Pure RNA buffer (Yekta Tajhiz Azma, Iran). After RNA treatment with DNase I (Thermo, USA), cDNA synthesis (Yekta Tajhiz Azma, Iran) was carried out based on the manufacturer’s instructions with random priming.

For measuring the relative expression of 5 major trophic factors including vascular endothelial growth factor (VEGF), neurotrophin-3 (NT-3), glial cell-derived neurotrophic factor (GDNF), brain-derived neurotrophic factor (BDNF), and nerve growth factor (NGF) as well as neuronal nuclei (NeuN, specific mature neuronal marker), quantitative real-time PCR technique was employed. The reactions contained first-strand cDNA template, specific primer sets (presented in Box 1), and SYBR Green Master Mix (AddBio, Korea) and were performed on Applied Biosystems StepOne with the following steps: 95.0 °C for 15 min, followed by 95.0° for 20 s and 60.0 °C for 1 min with a cycle number of 40. The data were normalized using the hypoxanthine-guanine phosphoribosyl transferase-1 [34] and the 2^−ΔΔCt^ method was used to calculate the fold change in the gene expression.

### Statistical analysis

GraphPad Prism (Version 7.5, GraphPad Software, Inc) was used for data analysis and presenting the data. Neurological scores were analyzed using a non-parametric Mann-Whitney test. Two-way analysis of variance (ANOVA) repeated measure followed by post hoc Tukey test was used to analyze body weight. Continuous variables were subjected to the Shapiro-Wilk normality test and comparisons between groups were made by one-way ANOVA followed by post hoc Tukey test or t-test. Data are shown as mean ± SEM or median ± interquartile interval and P < 0.05 was considered as statistically significant.

Box 1: Primer sequences (5′–3′) used in qPCR

**Table:** 

Gene	Forward primer (5′–3′)	Reverse primer (5′–3′)	Amplicon length (bp)
*BDNF*	CGATTAGGTGGCTTCATAGGAGAC	CAGAACAGAACAGAACAGAACAGG	182
*GDNF*	GCTGACCAGTGACTCCAATATGC	CCTCTGCGACCTTTCCCTCTG	192
*VEGF*	ACTTGAGTTGGGAGGAGGATGTC	GGATGGGTTTGTCGTGTTTCTGG	183
*NGF*	CCCAATAAAGGCTTTGCCAAGGAC	GAACAACATGGACATTACGCTATGC	78
*NT-3*	GACACAGAACTACTACGGCAACAG	ACTCTCCTCGGTGACTCTTATGC	184
*NeuN*	TGCTGAGATTTATGGAGGCTATGC	TGGTTCCGATGCTGTAGGTTGC	
*HPRT*	CCAGCGTCGTGATTAGTGATGATG	GAGCAAGTCTTTCAGTCCTGTCC	135

## Results

### Hair follicle stem cells isolation and characterization

**Fig. 1:**
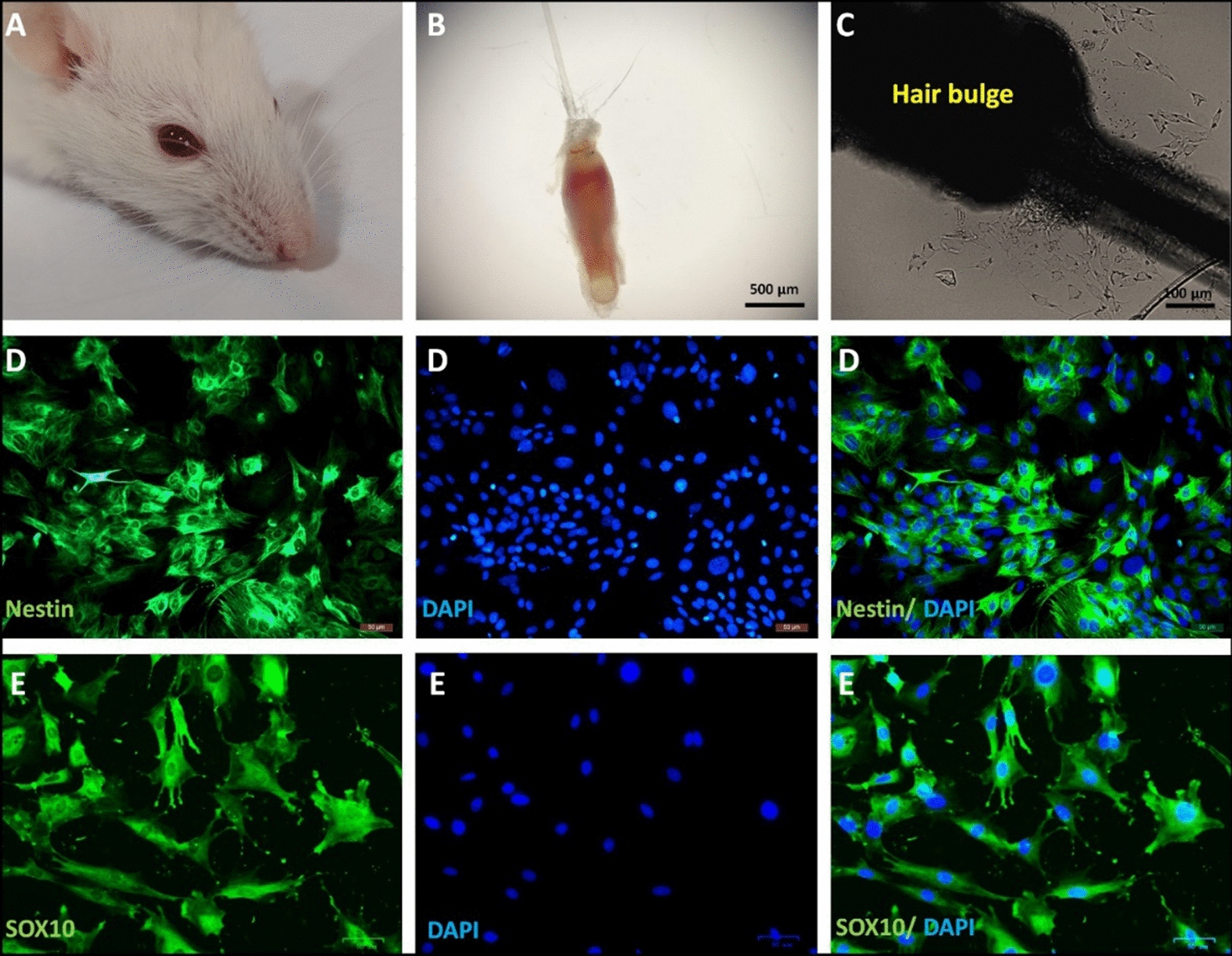
Isolation and verification of HFSCs. In this study, the whisker pad (**A**) and hair follicles of the rat (**B**) were dissected. The hair bulges were isolated and explanted in collagen-coated plates (**C**). After 2–3 days of explantation, migrated cells were observed around the bulge (**C**). The immunostaining demonstrated the nestin (**D**) and SOX10 (**E**) expression that verify the migrated cells as HFSCs

In the present study, hair follicles were obtained from the rat whisker pad (Fig. 1A and B) and bulges of hair follicles were explanted in collagen-coated wells. After a few days of culturing, migrated stem cells with stellate morphology were detected around the explanted bulges (Fig. 1C). Immunostaining revealed the high-level expression of neural crest stem cell marker—nestin—(Fig. 1D) and neural crest cell marker—SOX10—(Fig. 1E) by migrated stem cells which demonstrated the identity of migrated cells as HFSCs.

**Fig. 2:**
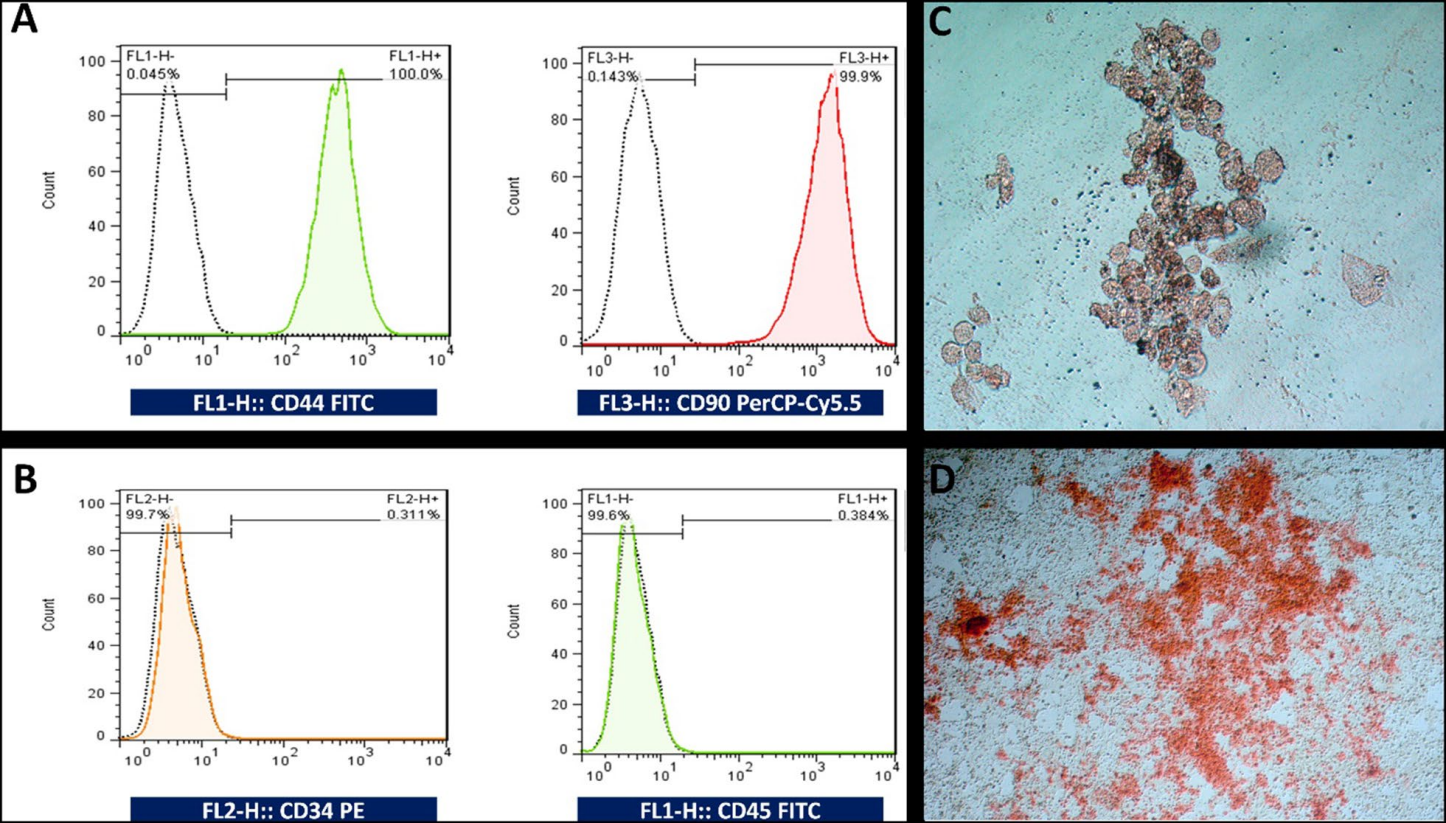
Characterization of stem cells. HFSCs were expressed CD44 and CD90 (**A**), but not CD34 and CD45 cell surface markers (**B**). HFSCs-cultured in adipogenic or osteogenic differentiation mediums followed by Oil Red (**C**) or Alizarin Red (**D**) staining’s

Furthermore, the flow cytometry analysis revealed in vitro cultured HFSCs were highly pure and expressed CD44 and CD90 as multipotent mesenchymal stem/stromal cell markers (Fig. 2A), but not CD34 and CD45 as hematopoietic markers (Fig. 2B). Moreover, Oil Red and Alizarin Red staining’s 21 days after sub-culturing in adipogenic and osteogenic differentiation mediums revealed that HFSCs were able to differentiate toward adipocytes (Fig. 2C) and osteoblasts (Fig. 2D), which showed their multipotency.

### Functional deficits

**Fig. 3:**
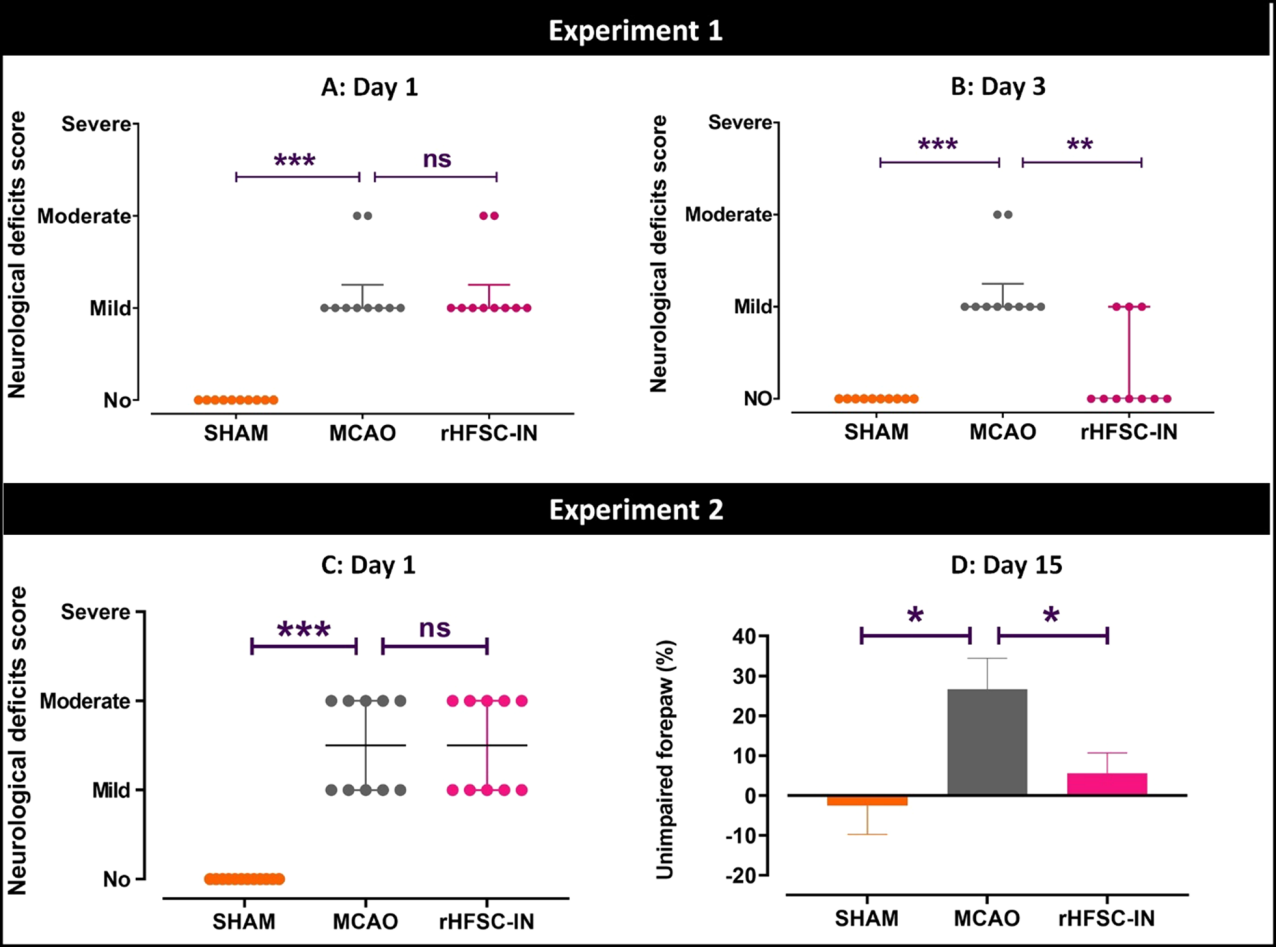
Neurological deficits. On days 1 (**A** and **C**) and 3 (**B**) after stroke, functional performance was evaluated by scoring method. On day 15 (**D**), preference for use of the impaired paw was determined by cylinder test. *P < 0.05, **P < 0.01, ***P < 0.001. *ns* not significant

In experiment 1, neurological outcome was evaluated before surgery (day 0) as well as 1 and 3 days post-ischemia/stem cell transplantation. On day 0, no impairment was found in the experimental animals (data not shown). One day post-surgery, the MCAO group represented remarkable functional deficits compared to the SHAM group (Fig. 3A). At this time point, the stem cell treated group showed neurological deficits very similar to the MCAO group (Fig. 3A). Interestingly, 3 days after cell therapy, the intranasal administration of HFSCs enhanced functional improvement compared to non-treated ischemic rats (Fig. 3B).

In experiment 2, functional performance was evaluated before surgery (day 0), before stem cell transplantation (day 1) and 14 days after the cell therapy. Again, no impairment was found on day 0 in the experimental animals (data not shown). Two hours MCA occlusion led to moderate to mild neurological deficits one day after stroke (Fig. 3 C). Also, MCAO induced a preference for the use of the unimpaired forepaw in the cylinder test when performed 15 days after stroke. Intranasal transplantation of HFSCs significantly decreased forepaw preference (Fig. 3D).

### Body weight and mortality rate

**Fig. 4:**
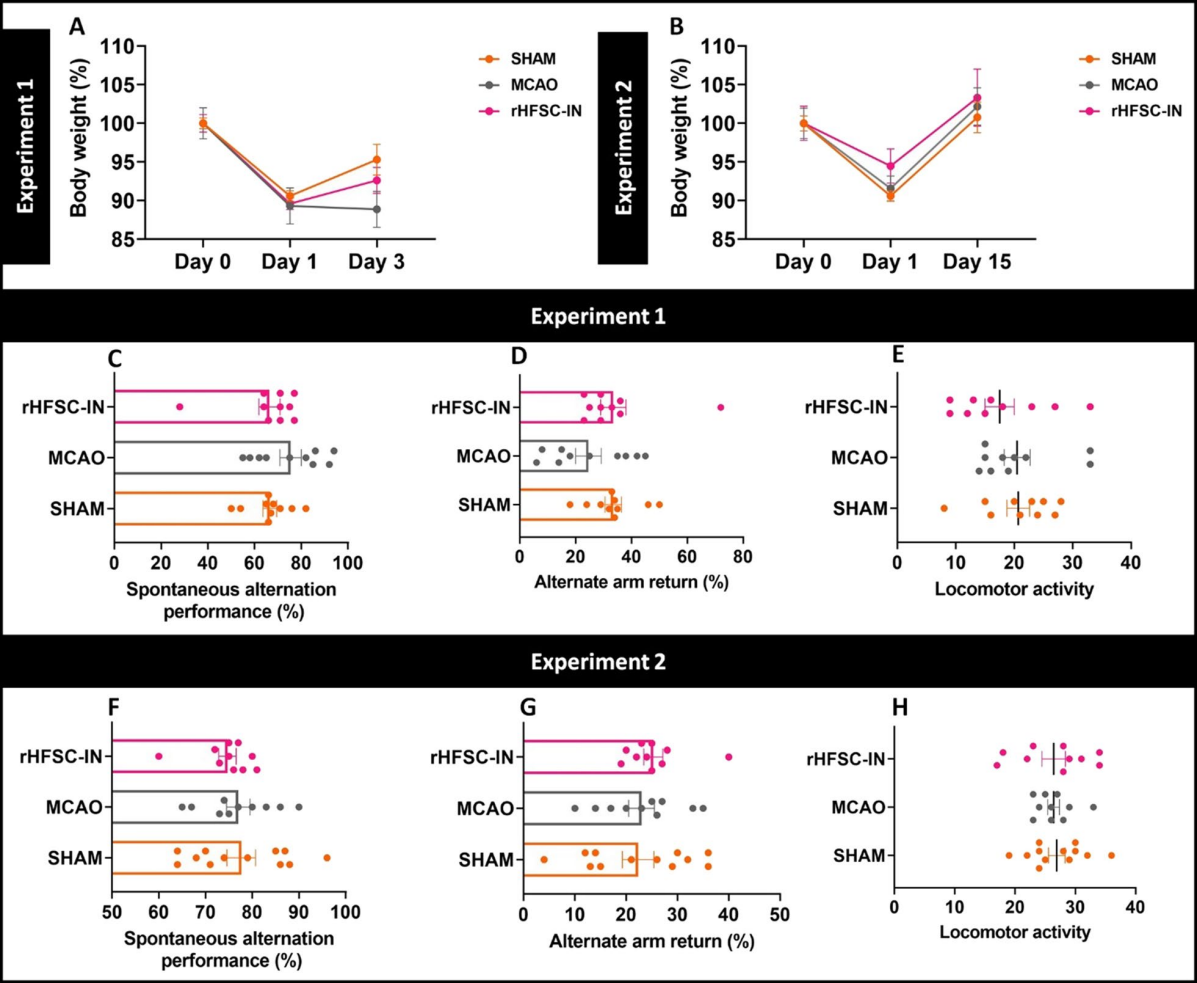
Body weight loss and spatial working memory. Body weight of animals in experimental groups at different time points (**A** and **B**). %SAP (**C** and **F**), %AAR (**D** and **G**) and locomotor activity (**E** and **H**) after 3 (experiment 1) or 15 days (experiment 2)

Body weight was assessed before surgery, one day after surgery and at the end point of both experiments (Fig. 4A and B). A considerable weight loss was observed in all rats that underwent surgery on day 1 in comparison with day 0. Although MCAO rats of experiment 1 did not gain weight, the body weight of SHAM and rHFSC-treated animals tended to increase until day 3 (Fig. 4A). All rats of experiment 2, gained weight on day 15 compared to day 1 (Fig. 4B). The mortality rate was also calculated during the experiment. According to our data, no mortality occurred in SHAM animals throughout the whole study. The mortality rate in the MCAO and HFSC-IN groups was ~ 20%.

### Short-term spatial memory

In the current experiment, the short-term spatial memory of animals following cerebral ischemia and stem cell transplantation was evaluated. Based on our findings, neither ischemia induction nor cell therapy could affect short-term spatial memory at days 3 and 15 as assessed by the Y-maze test. In this regard, there was no significant difference in the %SAP (Fig. 4C and F) and %AAR (Fig. 4D and G) between experimental groups. Furthermore, the total number of arm entries in the Y maze task as an index of locomotor activity was not affected by the interventions (Fig. 4E and H).

### Infarct volume

**Fig. 5:**
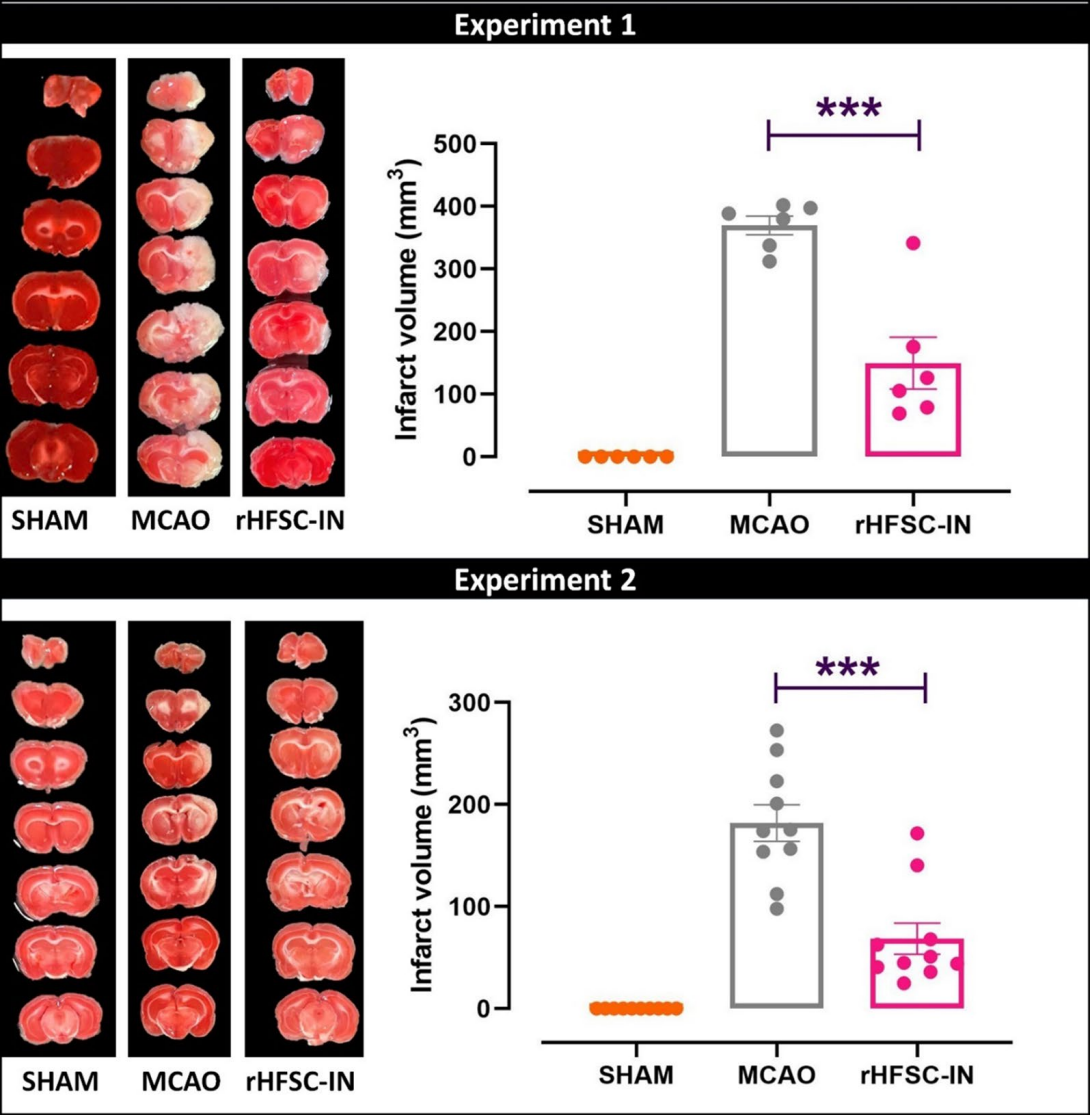
Infarct volume. Left panel: Coronal brain slices stained by TTC, three (experiment 1) or 14 (experiment 2) days after cell therapy. Right panel: Infarct volume expressed as mean ± SEM. ***P < 0.001

At the end point of both experiments, the infarct volume was evaluated by TTC staining (Fig. 5, Left panel). Obtained finding showed remarkable ischemic-induced damages in the ipsilateral hemisphere of the MCAO groups. The infarct volume significantly decreased in the stem cell-transplanted groups compared to the MCAO groups (Fig. 5, Right panel).

### Expression of target genes

**Fig. 6:**
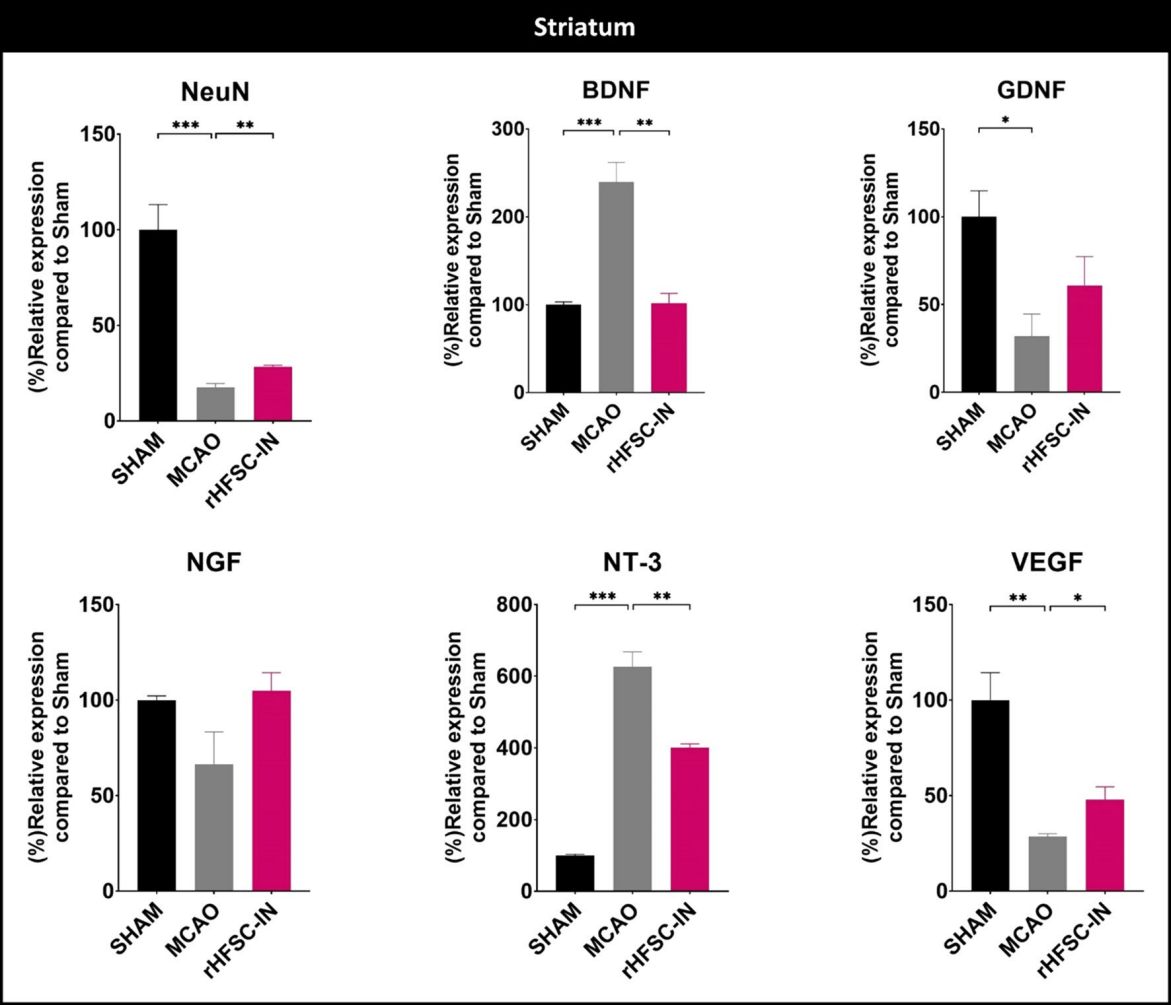
Relative expression of target genes in the ipsilateral striatum: relative expression of neuronal nuclei (NeuN), brain-derived neurotrophic factor (BDNF), glial cell-derived neurotrophic factor (GDNF), nerve growth factor (NGF), neurotrophin-3 (NT-3), and vascular endothelial growth factor (VEGF) were evaluated 3 days post-ischemia/cell administration in the striatum. *P < 0.05, **P < 0.01 and ***P < 0.001

In experiment 1, following 3 days of surgery/ stem cell therapy, relative expression of 5 main trophic factors, along with expression of NeuN as a mature neuron marker was assessed in the ipsilateral striatum and cortex. In the striatum, relative expression of GDNF and VEGF remarkably down-regulated following cerebral ischemia; while expression of NT-3 and BDNF was increased more than 600 and 200%, respectively. NGF expression was not statistically different between SHAM and MCAO groups. Ischemic stroke was also dramatically declined mature neuron biomarker expression, NeuN, at day 3 post-surgery. Cell therapy prevented over-expression of BDNF after stroke. Also, NT-3 transcript in the stem cell-treated group was lower than MCAO group. Moreover, a minor but significant increase in NeuN and VEGF levels was observed following intranasal delivery of HFSCs (Fig. 6).

**Fig. 7:**
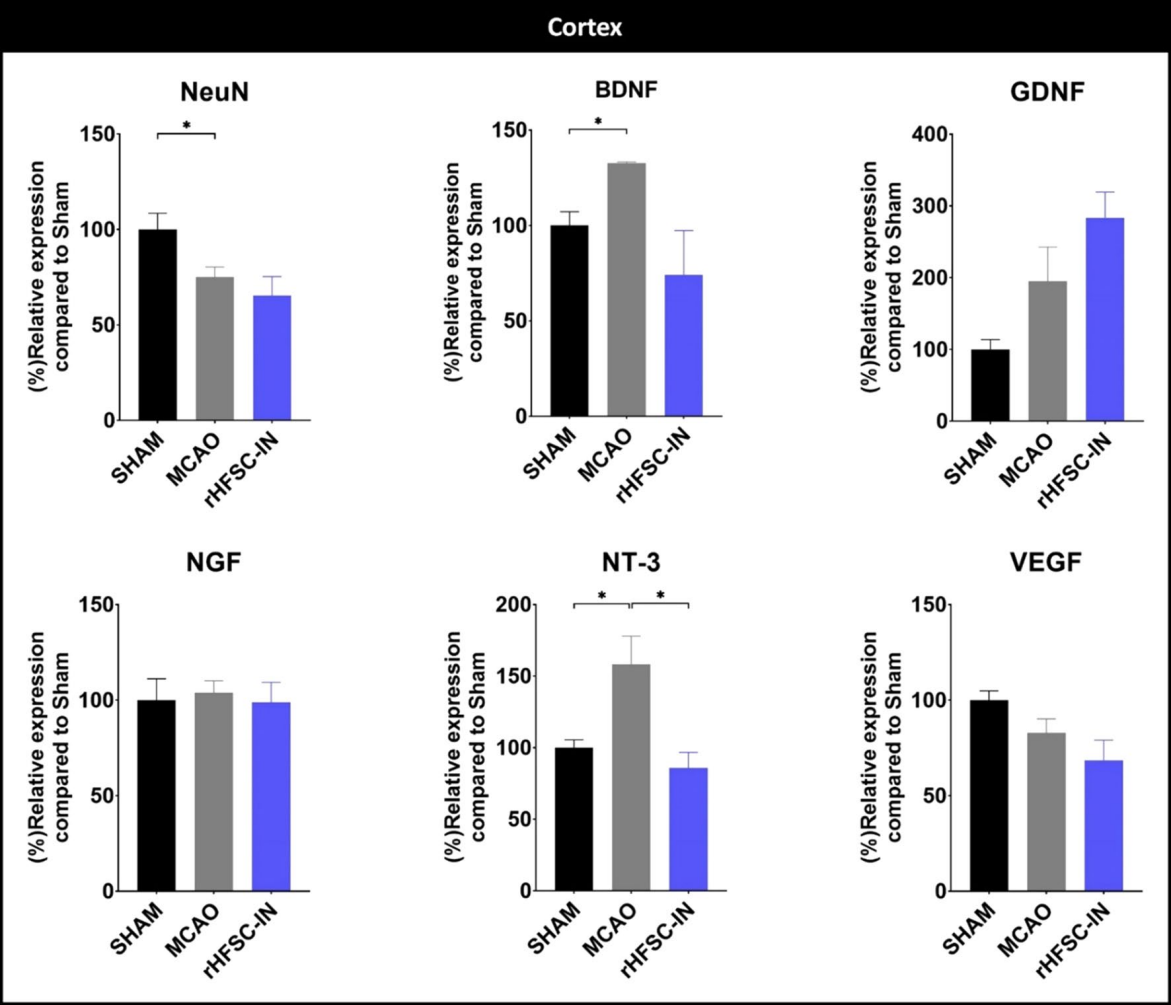
Relative expression of target genes in the ipsilateral cortex: relative expression of neuronal nuclei (NeuN), brain-derived neurotrophic factor (BDNF), glial cell-derived neurotrophic factor (GDNF), nerve growth factor (NGF), neurotrophin-3 (NT-3), and vascular endothelial growth factor (VEGF) were evaluated 3 days post-ischemia/cell administration in the cortex. *P < 0.05

In the cortex similar to the striatum, ischemic insult enhanced the relative expression of BDNF and NT-3 transcripts and rHFSC-IN prevented up-regulation of NT-3 mRNA. NeuN level in the cortex was also decreased after stroke; although stem cell transplantation was not able to restore it (Fig. 7).

## Discussion

A vast majority of pre-clinical investigations declare that stem cell transplantation can be considered as a promising strategy for the treatment of ischemic stroke. Up to now, the therapeutic potential of various stem cell types has been shown in animal models of brain ischemia [4]. Hence, using a stem cell type that can be easily harvested and cultured with a pure population is of paramount necessity. In addition, since stroke usually affects older patients, desired stem cells should preserve their multipotency in elderlies. HFSCs can be easily isolated from hairy skin and expanded in vitro with a highly pure population [35]. Also, it has been shown that neural crest stem cells obtained from the human skin of aged donors maintain their multipotency in vitro and in vivo [36]. Furthermore, HFSCs express a variety of trophic factors with neuroprotective, immunomodulatory and angiogenic potentials, and they can be easily manipulated by various preconditioning strategies [23, 37]. Therefore, HFSCs might be an invaluable asset in cell-based therapy for stroke.

Besides types of stem cells, the administration route is regarded among the critical factors that substantially affect the efficacy of cell therapy. Using non-neurosurgical pathways that can effectively pass the blood-brain barrier without systemic exposure is crucial for targeting the brain. For decades, the intranasal pathway has been employed to transport a variety of biologics to the brain for very different purposes [38]. In 2009, Danielyan and colleagues reported that bone-marrow mesenchymal stem cells reached the brain following intranasal administration. They proposed that cells migrate from the nasal mucosa through the cribriform plate, along the olfactory neural pathway into the brain. Accordingly, this path would eliminate or minimize the distribution of grafted cells into the peripheral organs [21]. In the last 10 years, several research groups investigated the therapeutic potential of intranasally-delivered stem cells in animal models of cerebral ischemia/hypoxia [39]; however, types of stem cells were limited to mesenchymal or neural stem cells [39].

Previously, we have shown that intra-arterial or intravenous administration of HFSCs immediately after 45 min MCAO led to a reduction in infarct size without significant effect on neurological impairment in rats. In addition, we found that cerebral expression of BDNF and NT-3 was altered, 7 days after the interventions [24]. Therefore, to expand our earlier findings, here in the present study, experimental animals underwent 30 or 120 min MCAO and received a single dose of HFSCs via the intranasal route. Stem cells were transplanted immediately after stroke induction or 24 h later. Neurological outcome impressively ameliorated in cerebral ischemic rats treated with HFSCs. In line with this data, the infarct volume was noticeably decreased after intranasal delivery of HFSCs. In addition, MCAO could not affect spatial working memory in the Y-maze task.

In animal models of stroke, the infarct size and neurological outcomes are considered as the essential parameters to evaluate the impairments associated with stroke and the efficacy of the treatment(s). Similar to our findings, Nijboer et al. reported that intranasal application of bone marrow mesenchymal stem cells in a rat model of subarachnoid hemorrhage, improved functional performance assessed by adhesive removal test. Also, myelin basic protein and microtubule-associated protein 2 staining’s revealed a significant reduction in brain tissue loss [40]. In addition, van Velthoven and colleagues transplanted mesenchymal stem cells via intranasal route, 3 days after 90 min MCAO in 10-day old male and female rats. Magnetic resonance imaging revealed a remarkable reduction in brain injury and cylinder rearing test showed an improvement in functional outcomes after the treatment [41].

In rats, striatum and cortex ipsilateral to the occlusion are always affected by transient MCAO [29]. Thus, in the present investigation, relative expression of six selected target genes was evaluated in the ipsilateral striatum and cortex, 3 days after MCAO/ cell therapy. In the striatum, the relative expression of BDNF and NT-3 was increased, while the expression of GDNF, VEGF, and NeuN was attenuated following MCAO. Intra-nasal administration of rHFSCs up-regulated the expression of NeuN and VEGF; while prevented over-expression of BDNF and NT-3 three days after the intervention. In the cortex, ischemic insult increased the expression of BDNF and NT-3 while it decreased the NeuN expression. Intranasal transplantation of HFSCs prevented over-expression of NT-3 in this region.

The NeuN, as a specific marker of mature neurons, is extensively expressed in the central nervous system and can be considered as an indicator of neuronal injuries [42]. It has been reported that reduction in cortical NeuN protein following MCAO, is connected with enhancement of lesion size evaluated by TTC staining [43]. Also, bone marrow mesenchymal stem cell transplantation resulted in an elevation of NeuN positive neurons in MCAO rats [44]. Here, we have found a significant reduction in NeuN expression in the both striatum and cortex of ischemic rats. NeuN expression in the striatum of HFSC-treated animals was slightly but significantly higher than non-treated MCAO rats; however, stem cell therapy remarkably decreased infarct volume assessed by TTC staining.

Growth factors are one of the most important regulators of various cellular processes such as neurogenesis, angiogenesis, cell proliferation, and differentiation. Since all of these processes are essential for recovery after stroke, numerous investigations employed exogenous growth factors to treat cerebral ischemia [45]. In addition, the beneficial potential of genetically engineered stem cells over-expressing growth factors has been demonstrated in the treatment of stroke [46]. It is documented that BDNF expression increased following different types of stroke such as intracerebral hemorrhage [47] and ischemic stroke [48]. It has been suggested that the BDNF expression pattern after stroke, agrees well with the neuroprotective effects of this neurotrophic factor [49]; nevertheless, the BDNF content in the lesioned hemisphere was not inversely correlated with neuronal death severity [50]. In line with these observations, we found a significant elevation of BDNF transcript in the brain regions. Interestingly, the BDNF mRNA level in the stem cell-treated group was at the SHAM level. A very similar expression-changing pattern was observed for the NT-3 transcript, a member of the neurotrophin family like BDNF; however, the expression of NGF, another member of the neurotrophin family was not statistically affected by the cerebral ischemia or stem cell transplantation.

GDNF is a well-known member of the GDNF family. Although the GDNF function has been substantially investigated, the underlying mechanism(s) for its secretion in pathological and physiological conditions is less known [51]. In earlier studies, Wei et al. in 2000 reported that GDNF expression at both transcription and translation levels elevated as early as 2 h after cerebral ischemia. Then, the expression declined and another enhancement was observed at 72 h [52]. In contrary, we found cerebral ischemia exerts dual opposite effects on the expression of GDNF in the striatum and cortex. Three days after 30 min occlusion, GDNF mRNA remarkably decreased in the striatum; while it tended to increase in the cortex. This pattern of GDNF expression was also observed in our previous experiment, 7 days after 45 min MCAO [24]. HFSCs administration through the intranasal route was not able to significantly alter the GDNF transcript compared to the MCAO group.

VEGF-A is a famous member of vascular endothelial growth factors and we referred to it as VEGF. This growth factor involves all phases of neovascularization including vasculogenesis, angiogenesis, arteriogenesis, also might exert neuroprotective effects in the brain. Thus, it is not surprising that VEGF plays a crucial role in the recovery after stroke [53]. Moreover, it has been reported that transplanted stem cells could exert curative impacts through enhancing VEGF-mediated repairing mechanisms and decreasing VEGF-mediated vascular leakage [54, 55]. However, induction of angiogenesis has dark sides and may increase chance of hemorrhagic transformation and edema that aggravate stroke outcomes [56, 57]. In the present study, we found that cerebral ischemia remarkably decreased VEGF expression especially in the striatum, and this reduction was partially compensated by HFSCs therapy. Also, we did not observe any sign of macroscopic hemorrhage.

## Conclusions

In the present study, we have shown that intranasal administration of HFSCs immediately after 30 min MCAO or one day after 120 min MCAO, improved functional performance that was accompanied by infarct volume reduction. At the moment, there is a lack of sufficient information regarding mechanisms underlying therapeutic actions of HFSCs following intranasal application. However, various potential mechanisms have been proposed for intranasally delivered stem cells such as stimulating endogenous repairing pathways, suppressing inflammatory processes, enhancing neurogenesis and angiogenesis, and possibly replacing damaged cells [32, 58]. Since the beneficial effects of HFSC therapy were observed from day 3 after transplantation, boosting endogenous regenerative pathways can be considered as one of the main mechanisms. Nevertheless, further investigations are required to clarify the exact mechanism(s). Moreover, in the current study cerebral ischemic rats received single shot of HFSCs; however, the intranasal route has a potential for repeated transplantation. Previously, it has been reported that repeated intranasal delivery of mesenchymal stem cells enhanced regeneration and functional performance after stroke in mice [59]. Therefore, the curative potential of repeated intranasal administration of HFSCs should also be assessed in the future.

## Data Availability

The datasets used and/or analyzed during the current study are available from the corresponding author on reasonable request.

## References

[1] Krishnamurthi RV, Ikeda T, Feigin VL (2020). Global, regional and country-specific burden of ischaemic stroke, intracerebral haemorrhage and subarachnoid haemorrhage: a systematic analysis of the global burden of disease study 2017. Neuroepidemiology.

[2] Kim JS (2019). tPA helpers in the treatment of acute ischemic stroke: are they ready for clinical use?. J Stroke.

[3] Singh M, Pandey PK, Bhasin A, Padma MV, Mohanty S (2020). Application of stem cells in stroke: a multifactorial approach. Front Neurosci.

[4] Tang YH, Ma YY, Zhang ZJ, Wang YT, Yang GY (2015). Opportunities and challenges: stem cell-based therapy for the treatment of ischemic stroke. CNS Neurosci Ther.

[5] Bang OY, Kim EH, Cha JM, Moon GJ (2016). Adult stem cell therapy for stroke: challenges and progress. J Stroke.

[6] Sieber-Blum M, Grim M, Hu Y, Szeder V (2004). Pluripotent neural crest stem cells in the adult hair follicle. Dev Dyn.

[7] Sakaue M, Sieber-Blum M (2015). Human epidermal neural crest stem cells as a source of Schwann cells. Development.

[8] Narytnyk A, Verdon B, Loughney A, Sweeney M, Clewes O, Taggart MJ (2014). Differentiation of human epidermal neural crest stem cells (hEPI-NCSC) into virtually homogenous populations of dopaminergic neurons. Stem Cell Rev Rep.

[9] Clewes O, Narytnyk A, Gillinder KR, Loughney AD, Murdoch AP, Sieber-Blum M (2011). Human epidermal neural crest stem cells (hEPI-NCSC)—characterization and directed differentiation into osteocytes and melanocytes. Stem Cell Rev Rep.

[10] Esmaeilzade B, Nobakht M, Joghataei MT, Roshandel NR, Rasouli H, Kuchaksaraei AS (2012). Delivery of epidermal neural crest stem cells (EPI-NCSC) to hippocamp in Alzheimer’s disease rat model. Iran Biomed J.

[11] Akbari S, Hooshmandi E, Bayat M, Borhani Haghighi A, Salehi MS, Pandamooz S (2022). The neuroprotective properties and therapeutic potential of epidermal neural crest stem cells transplantation in a rat model of vascular dementia. Brain Res.

[12] Li Y, Yao D, Zhang J, Liu B, Zhang L, Feng H (2017). The effects of epidermal neural crest stem cells on local inflammation microenvironment in the defected sciatic nerve of rats. Front Mol Neurosci.

[13] Hu YF, Gourab K, Wells C, Clewes O, Schmit BD, Sieber-Blum M (2010). Epidermal neural crest stem cell (EPI-NCSC)—mediated recovery of sensory function in a mouse model of spinal cord injury. Stem Cell Rev Rep.

[14] Pandamooz S, Salehi MS, Zibaii MI, Ahmadiani A, Nabiuni M, Dargahi L (2018). Epidermal neural crest stem cell-derived glia enhance neurotrophic elements in an ex vivo model of spinal cord injury. J Cell Biochem.

[15] Pandamooz S, Salehi MS, Nabiuni M, Dargahi L, Pourghasem M (2016). Evaluation of epidermal neural crest stem cells in organotypic spinal cord slice culture platform. Folia Biol..

[16] Sieber-Blum M (2010). Epidermal neural crest stem cells and their use in mouse models of spinal cord injury. Brain Res Bull.

[17] Rodriguez-Frutos B, Otero-Ortega L, Gutierrez-Fernandez M, Fuentes B, Ramos-Cejudo J, Diez-Tejedor E (2016). Stem cell therapy and administration routes after stroke. Transl Stroke Res.

[18] Fischer UM, Harting MT, Jimenez F, Monzon-Posadas WO, Xue H, Savitz SI (2009). Pulmonary passage is a major obstacle for intravenous stem cell delivery: the pulmonary first-pass effect. Stem Cells Dev.

[19] Guzman R, Janowski M, Walczak P (2018). Intra-arterial delivery of cell therapies for stroke. Stroke.

[20] Watanabe M, Yavagal DR (2016). Intra-arterial delivery of mesenchymal stem cells. Brain Circ.

[21] Danielyan L, Schäfer R, von Ameln-Mayerhofer A, Buadze M, Geisler J, Klopfer T (2009). Intranasal delivery of cells to the brain. Eur J Cell Biol.

[22] Zhang YT, He KJ, Zhang JB, Ma QH, Wang F, Liu CF (2021). Advances in intranasal application of stem cells in the treatment of central nervous system diseases. Stem Cell Res Ther.

[23] Pandamooz S, Jafari A, Salehi MS, Jurek B, Ahmadiani A, Safari A (2020). Substrate stiffness affects the morphology and gene expression of epidermal neural crest stem cells in a short term culture. Biotechnol Bioeng.

[24] Salehi MS, Pandamooz S, Safari A, Jurek B, Tamadon A, Namavar MR (2020). Epidermal neural crest stem cell transplantation as a promising therapeutic strategy for ischemic stroke. CNS Neurosci Ther.

[25] Karimi-Haghighi S, Chavoshinezhad S, Safari A, Razeghian-Jahromi I, Jamhiri I, Khodabandeh Z (2022). Preconditioning with secretome of neural crest-derived stem cells enhanced neurotrophic expression in mesenchymal stem cells. Neurosci Lett.

[26] Engel O, Kolodziej S, Dirnagl U, Prinz V (2011). Modeling stroke in mice-middle cerebral artery occlusion with the filament model. J Vis Exp.

[27] Longa EZ, Weinstein PR, Carlson S, Cummins R (1989). Reversible middle cerebral artery occlusion without craniectomy in rats. Stroke.

[28] Mousavi SM, Karimi-Haghighi S, Chavoshinezhad S, Pandamooz S, Belem-Filho IJA, Keshavarz S (2022). The impacts of anesthetic regimens on the middle cerebral artery occlusion outcomes in male rats. Neuroreport.

[29] Popp A, Jaenisch N, Witte OW, Frahm C (2009). Identification of ischemic regions in a rat model of stroke. PLoS ONE.

[30] Wei N, Yu SP, Gu X, Taylor TM, Song D, Liu X- (2013). Delayed intranasal delivery of hypoxic-preconditioned bone marrow mesenchymal stem cells enhanced cell homing and therapeutic benefits after ischemic stroke in mice. Cell Transplant.

[31] Magno LAV, Collodetti M, Tenza-Ferrer H, Romano-Silva MA (2019). Cylinder test to assess sensory-motor function in a mouse model of Parkinson’s disease. Bio Protoc.

[32] van Velthoven CT, Kavelaars A, van Bel F, Heijnen CJ (2010). Nasal administration of stem cells: a promising novel route to treat neonatal ischemic brain damage. Pediatr Res.

[33] Chavoshinezhad S, Kouchesfahani HM, Ahmadiani A, Dargahi L (2019). Interferon beta ameliorates cognitive dysfunction in a rat model of Alzheimer’s disease: modulation of hippocampal neurogenesis and apoptosis as underlying mechanism. Prog Neuropsychopharmacol Biol Psychiatry.

[34] Gubern C, Hurtado O, Rodríguez R, Morales JR, Romera VG, Moro MA (2009). Validation of housekeeping genes for quantitative real-time PCR in in-vivo and in-vitro models of cerebral ischaemia. BMC Mol Biol.

[35] Sieber-Blum M (2014). Human epidermal neural crest stem cells as candidates for cell-based therapies, disease modeling, and drug discovery. Birth Defects Res C Embryo Today.

[36] Moghadasi Boroujeni S, Koontz A, Tseropoulos G, Kerosuo L, Mehrotra P, Bajpai VK (2019). Neural crest stem cells from human epidermis of aged donors maintain their multipotency in vitro and in vivo. Sci Rep.

[37] Salehi MS, Borhani-Haghighi A, Pandamooz S, Safari A, Dargahi L, Dianatpour M (2019). Dimethyl fumarate up-regulates expression of major neurotrophic factors in the epidermal neural crest stem cells. Tissue Cell.

[38] Lochhead JJ, Thorne RG (2012). Intranasal delivery of biologics to the central nervous system. Adv Drug Deliv Rev.

[39] Salehi MS, Jurek B, Karimi-Haghighi S, Nezhad NJ, Mousavi SM, Hooshmandi E (2022). Intranasal application of stem cells and their derivatives as a new hope in the treatment of cerebral hypoxia/ischemia: a review. Rev Neurosci.

[40] Nijboer CH, Kooijman E, van Velthoven CT, van Tilborg E, Tiebosch IA, Eijkelkamp N (2018). Intranasal stem cell treatment as a novel therapy for subarachnoid hemorrhage. Stem Cells Dev.

[41] van Velthoven CT, Dzietko M, Wendland MF, Derugin N, Faustino J, Heijnen CJ (2017). Mesenchymal stem cells attenuate MRI-identifiable injury, protect white matter, and improve long-term functional outcomes after neonatal focal stroke in rats. J Neurosci Res.

[42] Gusel’nikova VV, Korzhevskiy DE (2015). NeuN as a neuronal nuclear antigen and neuron differentiation marker. Acta Naturae.

[43] Chen B, Lin W, Qi W, Li S, Hong Z, Zhao H (2020). Cofilin inhibition by limk1 reduces rod formation and cell apoptosis after ischemic stroke. Neuroscience.

[44] Xie P, Deng M, Sun QG, Ma YG, Zhou Y, Ming JH (2019). Therapeutic effect of transplantation of human bone marrow–derived mesenchymal stem cells on neuron regeneration in a rat model of middle cerebral artery occlusion. Mol Med Report.

[45] Lanfranconi S, Locatelli F, Corti S, Candelise L, Comi GP, Baron PL (2011). Growth factors in ischemic stroke. J Cell Mol Med.

[46] Salehi MS, Safari A, Pandamooz S, Jurek B, Hooshmandi E, Owjfard M (2022). The beneficial potential of genetically modified stem cells in the treatment of stroke: a review. Stem Cell Rev Rep.

[47] Guo YC, Song XK, Xu YF, Ma JB, Zhang JJ, Han PJ (2017). The expression and mechanism of BDNF and NGB in perihematomal tissue in rats with intracerebral hemorrhage. Eur Rev Med Pharmacol Sci.

[48] Grade S, Weng YC, Snapyan M, Kriz J, Malva JO, Saghatelyan A (2013). Brain-derived neurotrophic factor promotes vasculature-associated migration of neuronal precursors toward the ischemic striatum. PLoS One..

[49] Kokaia Z, Zhao Q, Kokaia M, Elmer E, Metsis M, Smith ML (1995). Regulation of brain-derived neurotrophic factor gene expression after transient middle cerebral artery occlusion with and without brain damage. Exp Neurol.

[50] Bejot Y, Prigent-Tessier A, Cachia C, Giroud M, Mossiat C, Bertrand N (2011). Time-dependent contribution of non neuronal cells to BDNF production after ischemic stroke in rats. Neurochem Int.

[51] Zhang Z, Sun GY, Ding S (2021). Glial cell line-derived neurotrophic factor and focal ischemic stroke. Neurochem Res.

[52] Wei G, Wu G, Cao X (2000). Dynamic expression of glial cell line-derived neurotrophic factor after cerebral ischemia. Neuroreport.

[53] Greenberg DA, Jin K (2013). Vascular endothelial growth factors (VEGFs) and stroke. Cell Mol Life Sci.

[54] Moon S, Chang MS, Koh SH, Choi YK (2021). Repair mechanisms of the neurovascular unit after ischemic stroke with a focus on VEGF. Int J Mol Sci.

[55] Harms KM, Li L, Cunningham LA (2010). Murine neural stem/progenitor cells protect neurons against ischemia by HIF-1alpha-regulated VEGF signaling. PLoS ONE.

[56] Adamczak J, Hoehn M (2015). Poststroke angiogenesis, con: dark side of angiogenesis. Stroke.

[57] Ergul A, Alhusban A, Fagan SC (2012). Angiogenesis: a harmonized target for recovery after stroke. Stroke.

[58] Donega V, Nijboer CH, Braccioli L, Slaper-Cortenbach I, Kavelaars A, van Bel F (2014). Intranasal administration of human MSC for ischemic brain injury in the mouse: in vitro and in vivo neuroregenerative functions. PLoS ONE.

[59] Chau MJ, Deveau TC, Gu X, Kim YS, Xu Y, Yu SP (2018). Delayed and repeated intranasal delivery of bone marrow stromal cells increases regeneration and functional recovery after ischemic stroke in mice. BMC Neurosci.

